# Vagus nerve stimulation in pediatric patients with drug-resistant epilepsy: a step-by-step video

**DOI:** 10.3171/2024.4.FOCVID244

**Published:** 2024-07-01

**Authors:** Santiago E. Cicutti, Guido P. Gromadzyn, Javier F. Cuello, Marcelo Bartuluchi

**Affiliations:** 1 Department of Neurosurgery, Juan P. Garrahan Hospital, Buenos Aires, Argentina; and; 2 Department of Neurosurgery, Hospital Provincial Petrona V. de Cordero, San Fernando, Buenos Aires, Argentina

**Keywords:** neurosurgery, vagus nerve stimulation, drug-resistant epilepsy

## Abstract

Vagus nerve stimulation (VNS) is a neuromodulatory treatment involving chronic intermittent electrical stimulation of the left vagus nerve, administered through a programmable pulse generator implanted subcutaneously in the chest. This generator connects to a bipolar lead, with electrodes wrapped around the vagus nerve in the neck. Primarily used as an adjunct therapy for patients with refractory epilepsy who cannot undergo or have not benefitted from resective surgery, VNS is generally well tolerated with few severe side effects. Herein is presented an educational surgical video providing a detailed, step-by-step technical description of VNS implantation.

The video can be found here: https://stream.cadmore.media/r10.3171/2024.4.FOCVID244

## Transcript

This video is a step-by-step description of the vagus nerve stimulator implantation technique, providing a general methodology and tips and tricks from an epilepsy surgeon with more than 20 years of experience.

### 0:33 Vagus Nerve Stimulation Overview.

Vagus nerve stimulation (VNS) is a neuromodulatory treatment that involves chronic intermittent electrical stimulation of the left vagus nerve, delivered by a programmable pulse generator. This pulse generator is subcutaneously implanted in the chest wall and connected to a bipolar lead with three helical coils wrapped around the cervical part of the vagus nerve.

### 0:55 Applications and Ideal Candidate of Vagus Nerve Stimulation.

VNS is used mainly for drug-resistant epilepsy, including focal and multifocal epilepsy, drop attacks, and syndromes like Lennox-Gastaut.

The ideal candidate for VNS implantation would be a patient suffering from a severe bilateral, nonlesional, drug-resistant epilepsy, where a resective or disconnective surgery wouldn’t be adequate. The main objective of the treatment is the reduction in the number and severity of these seizures, thus obtaining a better quality of life.^1–4^

### 1:29 VNS: Right- Versus Left-Side Implications.

The right vagus nerve innervates the sinoatrial node, and its stimulation could cause bradycardia, asystolia, and other cardiac side effects. Since the left vagus nerve innervates the atrioventricular node, the stimulator should be placed on the left side aiming to reduce cardiac side effects.

### 1:46 Surgical Technique: Patient Positioning.

The procedure is performed under general anesthesia, with endotracheal ventilatory support. Prophylactic antibiotics are administered 30 minutes before surgery. The patient is positioned supine on the operating room table with the head on a donut pillow or a headrest, extended and turned, approximately 30° to the right. The neck is extended with a shoulder roll in order to ease the passage of the tunneling rod.^1,5,6^

We have found that the most satisfactory approach in children is to begin by making both incisions in the direction of skin creases, which tends to minimize the amount of widening of the incision and gives a better overall cosmetic result. The skin of the left anterolateral neck and chest is prepared and draped in the usual sterile manner for surgery.

### 2:33 Neck Incision and Vagus Nerve Dissection.

A 2- to 3-cm transverse skin incision is made in a skin fold of the left side of the neck; the incision is carried out roughly halfway between the mastoid and the clavicle, extending from the midline to the medial border of the sternocleidomastoid muscle. The subcutaneous dissection exposes the platysma, which can be divided following the directions of its fibers. The omohyoid and other strap muscles are retracted medially, whereas the sternocleidomastoid is retracted laterally. Below this muscle plane, the dissection turns in a vertical direction, similar to the one of the carotid artery and jugular vein.

Once the carotid sheath is identified, it is opened sharply between the carotid artery medially and the internal jugular vein laterally. The vagus nerve is usually found in between these vessels and is dissected approximately 4 cm (avoiding damage to its vasa nervorum and preserving the perineurium sheath). The nerve is gently retracted superiorly using a vessel loop.

### 3:42 Chest Incision.

A 2.5- to 3-cm-long incision following a skin crease line is performed, approximately 5 cm below the clavicle and 5 cm medially to the shoulder. A 5-cm-diameter subcutaneous pocket is bluntly dissected, 5 cm below the clavicle or just medial to the axilla and superior to the breast.

### 4:04 Subcutaneous Tunneling.

The electrodes are tunneled with the manufacturer-supplied tunneling device, which includes an inner and an outer tunneling tube, and a bullet tip thread onto the end of the inner tube. To optimize the trajectory, the tunneler is slightly curved.

We choose to perform the tunneling from the neck incision toward the axillary incision. Once the track is created by the tunneling device, the bullet tip is removed and the shaft is withdrawn from the clear hollow sheath. The free end of the electrode is inserted in the sheath, and drawn, with the sheath, from the neck to the chest incision.

### 4:44 Simulator.

We have developed a simulator with the purpose of demonstrating the correct placement of the stimulator on the vagus nerve. The stimulator is equipped with three coils, the negative and positive leads, and the anchor or strain reliever. Each electrode is gently grasped one at a time for each pair of string-like attachments in the helices, and slightly stretched to facilitate the opening of the cleft between the helical turns.

The electrode is positioned on the right side, passing underneath the nerve. Once positioned beneath the nerve, the left hand [i.e., forceps] is used to reach the suture at the end of the helix, while the other left hand holds the opposite end of the suture on the right side of the nerve. Subsequently, controlled tension is applied to open the helices in the direction of the stimulator’s rotation, ensuring a precise fit on the nerve. The central turn of the spiral should be placed first, followed by the upper and lower turns that wrap around the nerve. The same steps are conducted with the rest of the helices.

### 5:53 Common Errors.

It is crucial to prevent the helices from being tangled upon themselves before placing the device, as this could significantly impede the electrode insertion. Therefore, it is imperative to stretch each helix and ensure it is correctly positioned before initiating the device placement.

Another common mistake is attempting to place the helices from the left side of the vagus nerve. Since the system is designed for right-handed surgeons, starting from the left side makes the placement very challenging (if not impossible). This simulator illustrates the difficulty of inserting the helices from the wrong position, contrasting with the ease and simplicity with which the device is placed from the right side of the nerve.

### 6:40 Electrode Placement.

During this step of the surgery, typically, the nerve is gently retracted upward with the vessel loop. Each of the electrodes is gently grasped and placed around the nerve. In applying each electrode, the central part of the spiral must be positioned first, and then the upper and lower turns are wound around the nerve. The tethering (inferior) anchor is secured around the vagus nerve, and then the positive (middle helical contact) and the negative (upper helical contact) electrodes are positioned.

To optimize the electrodes’ placement, we use a pair of tweezers to grasp the sutures at each end of the helix and pull outward, ensuring a proper stretch. As we explained before, starting with the distal electrode, we place the helix under the vagus nerve and pull its proximal end up to wrap it over the nerve. We repeat this process for the helix’s distal end, ensuring the electrode around the vagus nerve. These steps are applied to the middle and proximal electrodes. After the electrodes have been positioned on the nerve, it is necessary to make redundant loops so that movements of the neck do not dislodge the electrodes or put strain on the vagus nerve itself. The electrode is then secured by tacking nonabsorbable sutures to silicon holders at the deep cervical fascia.^1,5,6^

### 8:09 Connection of the Lead With the Pulse Generator.

With the pulse generator’s setscrew correctly positioned, the lead connector is fully inserted into the generator port. The connection is secured by tightening the setscrew clockwise with the torque wrench, ceasing rotation upon hearing a click. The excess lead is wrapped around the pulse generator and positioned into the subcutaneous pocket. The generator is sutured into place by stitching through the hole in the header onto the surrounding tissue using nonabsorbable sutures.

### 8:44 System Testing.

After implantation of the electrodes, the device is checked using the clinician programmer. A successful test result indicates the optimal functionality of both the implanted pulse generator, the lead, and the connections. The lead impedance is tested by a single stimulation impulse lasting 1 minute.

### 9:08 Wound Closure.

The wounds are closed in a standard fashion using absorbable sutures.

### 9:04 Postoperative.

The patient is normally discharged 1 day after surgery.

VNS is usually started 2 weeks after implantation, with recommended settings of stimulation that must be gradually reached, with 0.25-mA increases per week.

### 9:31 Complications.

The complications of VNS therapy are classified as early (related to surgery) and late (related to the device and nerve stimulation).^6–8^

### 9:41 VNS Lead Revision/Removal/Reimplantation.

In our pediatric VNS practice, we initially perform the neck incision as low as possible, anticipating the need for future device revisions. Our primary goal during revisions is to remove the entire device. However, if complete removal proves unfeasible, we carefully cut the electrode as close to the nerve as possible, and we then implant a new electrode proximally on the vagus nerve. This approach is designed to ensure minimal impact while maintaining effective treatment for our pediatric patients.

## Disclosures

The authors report no conflict of interest concerning the materials or methods used in this study or the findings specified in this publication.

## Author Contributions

Primary surgeon: Bartuluchi. Assistant surgeon: Cicutti. Editing and drafting the video and abstract: Cicutti, Cuello. Critically revising the work: all authors. Reviewed submitted version of the work: Cicutti, Cuello, Bartuluchi. Approved the final version of the work on behalf of all authors: Cicutti. Supervision: Gromadzyn, Cuello.

## Supplemental Information

### Patient Informed Consent

The necessary patient informed consent was obtained in this study.
